# Supplementary material for: A Likelihood Approach for Real-Time Calibration of Stochastic Compartmental Epidemic Models
Source: PLoS Comput Biol. 2017 Jan 17;13(1):e1005257. doi: 10.1371/journal.pcbi.1005257 (PMC5240920; doi:10.1371/journal.pcbi.1005257)
Supplement: S1 File — (TAR.GZ) [file pcbi.1005257.s014.tar.gz › HSPH_Online-SI-Revision/IPoi09/n10-extreme/n10-extreme_BENCH2_table4.pdf]

1	newly 3 weeks spec	135.	149.887	{71., 277.}	45.8184	0.167171	newly 3 weeks cumul	629.	679.387	{341., 1249.}	43.8074	0.164952	newly 5 weeks spec	61.	70.6613	{32., 138.}	50.1058	0.178751	newly 5 weeks cumul	793.	853.323	{421., 1582.}	44.7911	0.169484	newly 54 weeks cumul	895.	997.339	{481., 1854.}	45.3902	0.165116
2	newly 3 weeks spec	274.	296.82	{153., 460.}	43.2332	0.1694	newly 3 weeks cumul	1010.	1124.11	{653., 1673.}	38.3396	0.148258	newly 5 weeks spec	172.	185.131	{91., 297.}	46.6355	0.180187	newly 5 weeks cumul	1394.	1543.38	{878., 2344.}	40.2286	0.154824	newly 50 weeks cumul	1905.	2145.54	{1184., 3286.}	43.9852	0.163959
3	newly 3 weeks spec	87.	114.639	{53., 196.}	56.7929	0.180801	newly 3 weeks cumul	473.	603.623	{291., 1016.}	53.478	0.173101	newly 5 weeks spec	29.	39.9344	{16., 70.}	66.7609	0.210991	newly 5 weeks cumul	554.	712.197	{330., 1199.}	54.5363	0.175612	newly 56 weeks cumul	592.	774.525	{359., 1322.}	55.4663	0.17545
4	newly 3 weeks spec	199.	279.393	{144., 572.}	55.0457	0.159949	newly 3 weeks cumul	783.	1086.31	{597., 2117.}	51.4541	0.155001	newly 5 weeks spec	95.	162.787	{73., 353.}	80.2243	0.202676	newly 5 weeks cumul	1007.	1465.54	{758., 2895.}	57.0645	0.164384	newly 50 weeks cumul	1308.	1954.72	{978., 3968.}	61.1947	0.171007
5	newly 3 weeks spec	213.	106.71	{51., 189.}	59.0035	0.403104	newly 3 weeks cumul	916.	472.742	{230., 867.}	57.3778	0.383336	newly 5 weeks spec	132.	48.1452	{24., 92.}	65.7502	0.521356	newly 5 weeks cumul	1203.	593.403	{292., 1094.}	58.7201	0.401103	newly 53 weeks cumul	1516.	706.097	{349., 1365.}	59.9051	0.421803
6	newly 3 weeks spec	232.	221.41	{107., 405.}	47.117	0.204651	newly 3 weeks cumul	932.	995.279	{504., 1791.}	42.7672	0.166188	newly 5 weeks spec	93.	105.672	{47., 202.}	51.8068	0.189825	newly 5 weeks cumul	1166.	1254.46	{631., 2258.}	43.8799	0.168483	newly 54 weeks cumul	1415.	1474.69	{725., 2673.}	44.6261	0.177469
7	newly 3 weeks spec	196.	154.565	{81., 245.}	48.2143	0.241514	newly 3 weeks cumul	905.	717.145	{384., 1074.}	44.3646	0.219984	newly 5 weeks spec	97.	70.3226	{35., 98.}	52.3113	0.275774	newly 5 weeks cumul	1165.	891.435	{476., 1323.}	46.345	0.235218	newly 55 weeks cumul	1418.	1026.27	{527., 1526.}	48.6908	0.256903
8	newly 3 weeks spec	285.	183.516	{87., 288.}	51.2394	0.303339	newly 3 weeks cumul	1128.	755.726	{374., 1332.}	48.5115	0.279747	newly 5 weeks spec	172.	100.403	{45., 166.}	55.9171	0.351756	newly 5 weeks cumul	1506.	990.548	{490., 1699.}	49.6509	0.289467	newly 52 weeks cumul	1911.	1257.77	{617., 2161.}	50.4347	0.293924
9	newly 3 weeks spec	224.	244.21	{125., 432.}	43.138	0.163526	newly 3 weeks cumul	973.	997.823	{512., 1702.}	38.1776	0.156087	newly 5 weeks spec	131.	134.419	{63., 229.}	44.9643	0.181974	newly 5 weeks cumul	1278.	1315.76	{664., 2249.}	39.4051	0.159547	newly 52 weeks cumul	1632.	1683.39	{846., 2854.}	41.1132	0.165321
10	newly 3 weeks spec	311.	183.516	{87., 288.}	53.6666	0.33377	newly 3 weeks cumul	1154.	755.726	{374., 1332.}	49.0901	0.28709	newly 5 weeks spec	185.	100.403	{45., 166.}	57.7245	0.377581	newly 5 weeks cumul	1603.	990.548	{490., 1699.}	51.1581	0.309677	newly 52 weeks cumul	2165.	1257.77	{617., 2161.}	53.4903	0.335348
11	newly 3 weeks spec	280.	266.633	{115., 456.}	41.5357	0.191694	newly 3 weeks cumul	1095.	991.417	{456., 1679.}	37.9954	0.185037	newly 5 weeks spec	155.	178.65	{71., 309.}	54.7204	0.208835	newly 5 weeks cumul	1475.	1391.7	{633., 2353.}	39.4079	0.183773	newly 47 weeks cumul	1957.	2103.57	{872., 3422.}	48.4432	0.194311
12	newly 3 weeks spec	241.	449.115	{248., 854.}	88.7355	0.237007	newly 3 weeks cumul	1116.	1662.54	{981., 2861.}	53.8149	0.16895	newly 5 weeks spec	113.	279.393	{144., 572.}	147.512	0.3312	newly 5 weeks cumul	1420.	2299.74	{1330., 4198.}	65.8508	0.193005	newly 52 weeks cumul	1677.	3168.15	{1714., 6049.}	91.076	0.24072
13	newly 3 weeks spec	180.	364.286	{182., 652.}	103.086	0.257845	newly 3 weeks cumul	742.	1323.62	{750., 2221.}	79.8314	0.222247	newly 5 weeks spec	114.	234.508	{111., 420.}	108.187	0.261652	newly 5 weeks cumul	985.	1854.7	{982., 3121.}	89.4577	0.238931	newly 48 weeks cumul	1277.	2685.57	{1401., 4547.}	110.805	0.274398
14	newly 3 weeks spec	185.	151.887	{73., 267.}	47.2101	0.235582	newly 3 weeks cumul	825.	670.629	{327., 1267.}	45.0811	0.226321	newly 5 weeks spec	104.	72.5161	{36., 135.}	52.2953	0.297414	newly 5 weeks cumul	1066.	849.855	{417., 1591.}	46.1917	0.23522	newly 53 weeks cumul	1314.	1010.69	{501., 1954.}	47.4456	0.248353
15	newly 3 weeks spec	149.	241.918	{118., 499.}	70.8329	0.183197	newly 3 weeks cumul	603.	942.967	{502., 1848.}	64.6168	0.177734	newly 5 weeks spec	76.	145.049	{74., 324.}	94.1329	0.220356	newly 5 weeks cumul	785.	1274.67	{658., 2602.}	69.1239	0.182748	newly 50 weeks cumul	962.	1706.31	{861., 3497.}	80.8544	0.199127
16	newly 3 weeks spec	236.	286.77	{157., 517.}	42.3243	0.143808	newly 3 weeks cumul	954.	1201.75	{690., 2080.}	41.3909	0.137246	newly 5 weeks spec	131.	148.803	{72., 262.}	44.5501	0.162425	newly 5 weeks cumul	1261.	1556.89	{871., 2726.}	40.8432	0.136219	newly 53 weeks cumul	1555.	1908.95	{1040., 3347.}	41.8122	0.138837
17	newly 3 weeks spec	218.	226.806	{123., 352.}	40.8849	0.155033	newly 3 weeks cumul	974.	1032.52	{569., 1580.}	36.451	0.139448	newly 5 weeks spec	107.	103.968	{53., 152.}	45.6437	0.185734	newly 5 weeks cumul	1233.	1291.05	{704., 1977.}	37.6985	0.14481	newly 55 weeks cumul	1481.	1496.21	{795., 2275.}	38.5452	0.152547
18	newly 3 weeks spec	202.	267.387	{139., 406.}	52.5072	0.157553	newly 3 weeks cumul	887.	1108.18	{595., 1831.}	45.3486	0.148198	newly 5 weeks spec	107.	137.371	{64., 227.}	57.5671	0.181579	newly 5 weeks cumul	1132.	1435.9	{755., 2335.}	47.5037	0.15156	newly 53 weeks cumul	1431.	1769.26	{901., 2912.}	48.2361	0.157071
19	newly 3 weeks spec	155.	226.806	{123., 352.}	56.7118	0.154889	newly 3 weeks cumul	791.	1032.52	{569., 1580.}	44.0683	0.136895	newly 5 weeks spec	62.	103.968	{53., 152.}	74.6098	0.18292	newly 5 weeks cumul	957.	1291.05	{704., 1977.}	47.4618	0.141639	newly 55 weeks cumul	1067.	1496.21	{795., 2275.}	51.1156	0.14622
20	newly 3 weeks spec	277.	338.227	{164., 590.}	44.4958	0.159488	newly 3 weeks cumul	1115.	1346.95	{679., 2268.}	40.8897	0.150308	newly 5 weeks spec	154.	190.773	{90., 344.}	48.9524	0.170437	newly 5 weeks cumul	1485.	1795.35	{893., 3052.}	42.2806	0.154227	newly 52 weeks cumul	1930.	2317.05	{1156., 3962.}	43.3693	0.15821
21	newly 3 weeks spec	230.	283.65	{149., 467.}	47.413	0.159801	newly 3 weeks cumul	931.	1051.4	{590., 1725.}	37.927	0.143113	newly 5 weeks spec	137.	184.883	{80., 297.}	60.3528	0.177589	newly 5 weeks cumul	1250.	1466.88	{789., 2407.}	42.1667	0.15061	newly 49 weeks cumul	1622.	2109.9	{1052., 3385.}	51.942	0.162292
22	newly 3 weeks spec	126.	175.05	{92., 243.}	55.3307	0.166274	newly 3 weeks cumul	596.	875.1	{469., 1240.}	55.9732	0.160874	newly 5 weeks spec	39.	68.7167	{31., 106.}	83.8889	0.218384	newly 5 weeks cumul	702.	1055.73	{570., 1511.}	58.9079	0.166626	newly 56 weeks cumul	785.	1170.83	{624., 1670.}	58.7601	0.167335
23	newly 3 weeks spec	183.	279.393	{144., 572.}	62.7609	0.172114	newly 3 weeks cumul	736.	1086.31	{597., 2117.}	57.2768	0.165708	newly 5 weeks spec	84.	162.787	{73., 353.}	98.79	0.235642	newly 5 weeks cumul	938.	1465.54	{758., 2895.}	64.7978	0.179241	newly 51 weeks cumul	1140.	1954.72	{978., 3968.}	78.1334	0.203257
24	newly 3 weeks spec	218.	234.131	{113., 374.}	45.9016	0.180725	newly 3 weeks cumul	857.	898.41	{496., 1381.}	41.0161	0.166686	newly 5 weeks spec	150.	143.689	{73., 249.}	46.9617	0.203722	newly 5 weeks cumul	1186.	1227.23	{676., 1927.}	42.1516	0.172025	newly 49 weeks cumul	1579.	1685.7	{898., 2620.}	45.4812	0.178118
25	newly 3 weeks spec	123.	326.066	{154., 562.}	165.574	0.368706	newly 3 weeks cumul	613.	1287.84	{679., 2171.}	111.013	0.284381	newly 5 weeks spec	53.	183.115	{87., 326.}	245.5	0.47103	newly 5 weeks cumul	719.	1720.39	{888., 2955.}	139.764	0.33327	newly 52 weeks cumul	805.	2218.21	{1105., 3848.}	175.681	0.386474
26	newly 3 weeks spec	82.	156.21	{82., 259.}	93.0173	0.232065	newly 3 weeks cumul	370.	747.	{389., 1258.}	102.833	0.257374	newly 5 weeks spec	10.	63.2258	{31., 112.}	532.258	0.733614	newly 5 weeks cumul	409.	911.532	{462., 1528.}	123.129	0.295372	newly 55 weeks cumul	439.	1030.08	{520., 1721.}	134.804	0.315913
27	newly 3 weeks spec	253.	286.77	{157., 517.}	39.3831	0.143333	newly 3 weeks cumul	1055.	1201.75	{690., 2080.}	36.4976	0.132899	newly 5 weeks spec	136.	148.803	{72., 262.}	43.6234	0.16557	newly 5 weeks cumul	1391.	1556.89	{871., 2726.}	36.8634	0.135405	newly 53 weeks cumul	1730.	1908.95	{1040., 3347.}	38.1673	0.141402
28	newly 3 weeks spec	143.	68.6393	{27., 112.}	60.2774	0.427693	newly 3 weeks cumul	714.	367.41	{168., 623.}	57.8271	0.393014	newly 5 weeks spec	59.	24.0492	{9., 39.}	64.2956	0.498843	newly 5 weeks cumul	874.	431.393	{197., 736.}	58.9395	0.409178	newly 56 weeks cumul	975.	469.672	{210., 822.}	59.4149	0.417576
29	newly 3 weeks spec	161.	357.803	{179., 691.}	123.195	0.293799	newly 3 weeks cumul	747.	1363.98	{759., 2573.}	85.0528	0.231505	newly 5 weeks spec	83.	216.443	{101., 425.}	160.774	0.341979	newly 5 weeks cumul	974.	1859.82	{1002., 3570.}	93.101	0.243352	newly 51 weeks cumul	1110.	2511.79	{1298., 4996.}	126.766	0.301086
30	newly 3 weeks spec	187.	279.393	{144., 572.}	60.4716	0.167957	newly 3 weeks cumul	802.	1086.31	{597., 2117.}	49.4358	0.151487	newly 5 weeks spec	78.	162.787	{73., 353.}	111.854	0.25852	newly 5 weeks cumul	1008.	1465.54	{758., 2895.}	56.9737	0.164236	newly 51 weeks cumul	1234.	1954.72	{978., 3968.}	67.6781	0.182899
31	newly 3 weeks spec	252.	216.984	{107., 403.}	46.5758	0.22115	newly 3 weeks cumul	969.	976.742	{504., 1791.}	41.4861	0.170322	newly 5 weeks spec	111.	103.274	{45., 190.}	48.9247	0.219825	newly 5 weeks cumul	1242.	1229.9	{631., 2258.}	42.629	0.177719	newly 54 weeks cumul	1508.	1444.58	{725., 2673.}	43.4928	0.187516
32	newly 3 weeks spec	205.	318.776	{157., 573.}	68.3852	0.206901	newly 3 weeks cumul	891.	1142.28	{621., 1870.}	48.1946	0.173312	newly 5 weeks spec	111.	229.	{90., 409.}	116.278	0.280845	newly 5 weeks cumul	1156.	1637.43	{845., 2707.}	58.2344	0.191685	newly 46 weeks cumul	1578.	2631.26	{1176., 4593.}	82.1238	0.228867
33	newly 3 weeks spec	80.	154.565	{81., 245.}	96.9556	0.232569	newly 3 weeks cumul	415.	717.145	{384., 1074.}	75.892	0.194408	newly 5 weeks spec	25.	70.3226	{35., 98.}	181.29	0.36837	newly 5 weeks cumul	481.	891.435	{476., 1323.}	87.0934	0.215371	newly 54 weeks cumul	518.	1026.27	{527., 1526.}	98.9382	0.237394
34	newly 3 weeks spec	107.	68.6393	{27., 112.}	53.0106	0.324847	newly 3 weeks cumul	518.	367.41	{168., 623.}	50.8197	0.287464	newly 5 weeks spec	37.	24.0492	{9., 39.}	51.9273	0.327647	newly 5 weeks cumul	629.	431.393	{197., 736.}	51.3175	0.297782	newly 56 weeks cumul	672.	469.672	{210., 822.}	51.0587	0.292633
35	newly 3 weeks spec	219.	216.443	{101., 425.}	48.2371	0.203085	newly 3 weeks cumul	914.	853.639	{434., 1688.}	44.0937	0.196171	newly 5 weeks spec	143.	125.344	{59., 259.}	51.8056	0.244163	newly 5 weeks cumul	1246.	1141.77	{564., 2300.}	45.6096	0.205899	newly 51 weeks cumul	1672.	1505.61	{709., 3114.}	47.3429	0.217448
36	newly 3 weeks spec	304.	283.65	{149., 467.}	40.6853	0.182339	newly 3 weeks cumul	1147.	1051.4	{590., 1725.}	34.1296	0.158463	newly 5 weeks spec	161.	184.883	{80., 297.}	52.9089	0.184613	newly 5 weeks cumul	1520.	1466.88	{789., 2407.}	37.4463	0.163254	newly 49 weeks cumul	2076.	2109.9	{1052., 3385.}	43.0684	0.173491
37	newly 3 weeks spec	209.	328.	{165., 577.}	65.2724	0.183958	newly 3 weeks cumul	875.	1287.84	{684., 2170.}	56.0369	0.169945	newly 5 weeks spec	117.	183.516	{87., 288.}	67.9901	0.184743	newly 5 weeks cumul	1151.	1715.56	{905., 2925.}	58.2004	0.17228	newly 52 weeks cumul	1404.	2217.61	{1162., 3754.}	65.6006	0.184234
38	newly 3 weeks spec	55.	156.21	{82., 259.}	184.018	0.39336	newly 3 weeks cumul	284.	747.	{389., 1258.}	163.028	0.367937	newly 5 weeks spec	18.	63.2258	{31., 112.}	251.254	0.478342	newly 5 weeks cumul	331.	911.532	{462., 1528.}	175.387	0.386089	newly 55 weeks cumul	350.	1030.08	{520., 1721.}	194.309	0.413589
39	newly 3 weeks spec	107.	156.21	{82., 259.}	59.5568	0.172918	newly 3 weeks cumul	564.	747.	{389., 1258.}	48.2899	0.151323	newly 5 weeks spec	42.	63.2258	{31., 112.}	63.3641	0.177094	newly 5 weeks cumul	669.	911.532	{462., 1528.}	50.8872	0.155302	newly 55 weeks cumul	739.	1030.08	{520., 1721.}	52.881	0.15798
40	newly 3 weeks spec	222.	328.	{165., 577.}	58.6167	0.171263	newly 3 weeks cumul	944.	1287.84	{684., 2170.}	48.8621	0.15602	newly 5 weeks spec	115.	183.516	{87., 288.}	69.8738	0.187764	newly 5 weeks cumul	1200.	1715.56	{905., 2925.}	53.9906	0.16415	newly 52 weeks cumul	1524.	2217.61	{1162., 3754.}	56.9406	0.168378
41	newly 3 weeks spec	212.	279.393	{144., 572.}	50.8429	0.15622	newly 3 weeks cumul	904.	1086.31	{597., 2117.}	43.3501	0.148948	newly 5 weeks spec	89.	162.787	{73., 353.}	89.5745	0.219249	newly 5 weeks cumul	1155.	1465.54	{758., 2895.}	46.9818	0.151232	newly 51 weeks cumul	1447.	1954.72	{978., 3968.}	52.5757	0.158415
42	newly 3 weeks spec	235.	266.633	{115., 456.}	46.156	0.178655	newly 3 weeks cumul	936.	991.417	{456., 1679.}	40.4754	0.16932	newly 5 weeks spec	165.	178.65	{71., 309.}	52.1111	0.211996	newly 5 weeks cumul	1287.	1391.7	{633., 2353.}	42.331	0.172441	newly 46 weeks cumul	1777.	2103.57	{872., 3422.}	51.9509	0.188559
43	newly 3 weeks spec	116.	227.048	{111., 346.}	98.6235	0.234087	newly 3 weeks cumul	596.	1061.23	{536., 1583.}	82.664	0.215748	newly 5 weeks spec	40.	96.1452	{39., 144.}	143.75	0.302369	newly 5 weeks cumul	704.	1304.53	{636., 1948.}	89.4176	0.226195	newly 56 weeks cumul	757.	1473.56	{697., 2214.}	98.1229	0.241606
44	newly 3 weeks spec	281.	296.571	{149., 508.}	42.5295	0.173646	newly 3 weeks cumul	1071.	1102.52	{573., 1908.}	39.4736	0.166434	newly 5 weeks spec	173.	187.54	{84., 363.}	50.4817	0.199158	newly 5 weeks cumul	1453.	1524.57	{760., 2678.}	41.815	0.172142	newly 47 weeks cumul	1985.	2167.9	{1083., 3741.}	45.2609	0.177519
45	newly 3 weeks spec	250.	357.803	{179., 691.}	55.4885	0.162818	newly 3 weeks cumul	978.	1363.98	{759., 2573.}	49.4871	0.153964	newly 5 weeks spec	142.	216.443	{101., 425.}	66.1856	0.180092	newly 5 weeks cumul	1328.	1859.82	{1002., 3570.}	51.3122	0.156227	newly 51 weeks cumul	1725.	2511.79	{1298., 4996.}	56.8401	0.165133
46	newly 3 weeks spec	267.	114.117	{49., 179.}	68.5331	0.506968	newly 3 weeks cumul	1078.	446.167	{201., 727.}	67.4892	0.504108	newly 5 weeks spec	173.	67.8333	{27., 111.}	72.2929	0.563708	newly 5 weeks cumul	1493.	603.35	{256., 965.}	68.7464	0.520068	newly 49 weeks cumul	2102.	827.9	{337., 1275.}	70.4408	0.540596
47	newly 3 weeks spec	255.	357.803	{179., 691.}	53.8798	0.160533	newly 3 weeks cumul	1074.	1363.98	{759., 2573.}	42.1635	0.140735	newly 5 weeks spec	129.	216.443	{101., 425.}	77.2906	0.198484	newly 5 weeks cumul	1366.	1859.82	{1002., 3570.}	48.9271	0.152009	newly 51 weeks cumul	1768.	2511.79	{1298., 4996.}	54.6056	0.161386
48	newly 3 weeks spec	231.	304.597	{160., 589.}	48.6315	0.151861	newly 3 weeks cumul	980.	1289.05	{708., 2286.}	45.051	0.143101	newly 5 weeks spec	109.	151.887	{73., 267.}	56.1261	0.165631	newly 5 weeks cumul	1224.	1655.08	{887., 2964.}	48.1881	0.148582	newly 54 weeks cumul	1459.	1995.15	{1052., 3651.}	49.9403	0.151577
49	newly 3 weeks spec	157.	226.806	{123., 352.}	55.5373	0.152913	newly 3 weeks cumul	774.	1032.52	{569., 1580.}	45.7739	0.140066	newly 5 weeks spec	60.	103.968	{53., 152.}	78.871	0.190501	newly 5 weeks cumul	908.	1291.05	{704., 1977.}	51.9593	0.149839	newly 55 weeks cumul	1013.	1496.21	{795., 2275.}	56.0217	0.155456
50	newly 3 weeks spec	221.	340.258	{173., 585.}	64.8227	0.186529	newly 3 weeks cumul	1020.	1391.47	{724., 2289.}	50.5708	0.163424	newly 5 weeks spec	108.	178.532	{84., 301.}	75.5227	0.202511	newly 5 weeks cumul	1290.	1817.89	{926., 3027.}	54.1723	0.16908	newly 53 weeks cumul	1505.	2251.34	{1111., 3747.}	60.7405	0.17963
